# Antibody-Drug Conjugates: Functional Principles and Applications in Oncology and Beyond

**DOI:** 10.3390/vaccines9101111

**Published:** 2021-09-29

**Authors:** Charalampos Theocharopoulos, Panagiotis-Petros Lialios, Michael Samarkos, Helen Gogas, Dimitrios C. Ziogas

**Affiliations:** First Department of Medicine, School of Medicine, National and Kapodistrian University of Athens, Laiko General Hospital, 115 27 Athens, Greece; hartheoch@gmail.com (C.T.); lialiospeter@gmail.com (P.-P.L.); msamarkos@gmail.com (M.S.); helgogas@gmail.com (H.G.)

**Keywords:** antibody-drug conjugates, immunoconjugates, bispecific antibodies, dual-drug ADCs, targeted therapy

## Abstract

In the era of precision medicine, antibody-based therapeutics are rapidly enriched with emerging advances and new proof-of-concept formats. In this context, antibody-drug conjugates (ADCs) have evolved to merge the high selectivity and specificity of monoclonal antibodies (mAbs) with the cytotoxic potency of attached payloads. So far, ten ADCs have been approved by FDA for oncological indications and many others are currently being tested in clinical and preclinical level. This paper summarizes the essential components of ADCs, from their functional principles and structure up to their limitations and resistance mechanisms, focusing on all latest bioengineering breakthroughs such as bispecific mAbs, dual-drug platforms as well as novel linkers and conjugation chemistries. In continuation of our recent review on anticancer implication of ADC’s technology, further insights regarding their potential usage outside of the oncological spectrum are also presented. Better understanding of immunoconjugates could maximize their efficacy and optimize their safety, extending their use in everyday clinical practice.

## 1. Introduction

**Figure 1:**
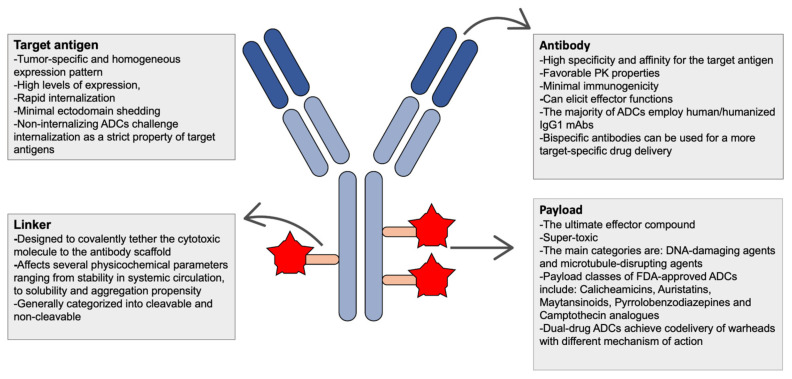
Simplified design and favorable characteristics of ADCs’ components.

Antibody-drug conjugates (ADCs) comprise a fast-expanding therapeutic modality designed to target disease cells, sparing the adjacent healthy tissues. The regulatory approval of first-generation ADCs spurred a surge of interest in this biotechnology that has produced a total of ten FDA-approved agents and an ever-increasing panel of candidates in clinical and preclinical level. ADCs are essentially tripartite pro-drugs consisting of an antibody tethered via a chemical linker to a given payload [1]. (Figure 1) After their administration, these agents circulate as inactive assemblies which are eventually catabolized via endogenous cleavage mechanisms at the intracellular compartment of the targeted cell [2]. Exploiting the embedded properties of monoclonal antibodies (mAbs), these immunoconjugates achieve selective delivery and localized release of the attached payload, minimizing the “off-target” effects on normal tissues and improving the therapeutic index [3]. The clinical utility of ADCs has been mainly explored in hematological/oncological indications [1]. However, during the last decade, a constant flow of reports about the implementation of this technology outside the oncological sphere has been also observed. In fact, brentuximab vedotin (BV), ABBV-3373, and DSTA4637S are currently under clinical testing for non-oncological indications, such as autoimmune and infectious diseases [4,5,6]. The discrepancy between the initially large number of ADCs in pharmaceutical pipelines and the small proportion of agents reaching late-stage trials highlights the need for deeper understanding, meticulous selection, and constant optimization of ADC components. In the following sections, we elaborate on the functional principles and the characteristics of these components (Box 1), with emphasis on the latest bioengineering advances, such as bispecific antibodies, multi-drug ADCs, non-internalizing ADCs, and ADC-antibody co-administration. Updating our previous report [1], all current oncological and non-oncological implications of ADC’s technology are also recapped.

Box 1 **Essential components and considerations in the design of ADCs.**

**Essential components of ADCS**

**1** 
**ANTIBODIES**


**Types**

**Considerations for Abs**

**Considerations for target antigen**

Monoclonal Ab (chimeric, humanized or human, mainly IgG1 isotype)Bispecific Ab

Specificity and affinity for the target antigenInternalizationPharmacokinetic propertiesEffector functionsImmunogenicity

Tumour-specificityInternalizationExpression patternEctodomain shedding

**2** 
**LINKERS**


**Types**

**Considerations for linker**

**Considerations for conjugation**

Cleavable linkersNon-cleavable linkers

Circulation stabilitySolubilityAggregation propensity

Drug to antibody ratio (DAR)Homogeneity

**3** 
**PAYLOADS**


**Types**

**Considerations for payload**

**Mechanisms of drug resistance**

CalicheamicinsPyrrolobenzodiazepines (PBDs)Auristatins Maytansinoids Camptothecin (CPT)

Half lifeBystander killing activitySystemic accumulationPotential for resistance development

Antigen-related resistanceDeficient lysosomal functionUpregulated efflux pumpsSurvival/apoptotic signalingBSB phenomenon

## 2. Functional Principles and Essential Components of ADCs

### 2.1. Antigen Selection and Antibodies

The fundamental step of ADC development remains the selection of target-antigen [7,8]. The optimal target-antigen should be characterized by tumor-specific and homogeneous expression pattern, high levels of expression, rapid internalization, and minimal ectodomain shedding. The exclusive expression of an epitope on the cancer cell surface represents an ideal scenario, since most selected antigens are, in fact, tumor-associated and not tumor-restricted [9]. In general, it is preferable for these tumor-associated antigens to be localized either on tissues resistant to the given payload or on tissues with high regenerative capacity [10]. The level of antigen expression critically affects the therapeutic index of the immunoconjugate as it defines the amount of the cytotoxic payload that will be internalized in the cancer cell [11]. In solid tumor cell lines, this correlation between surface antigen density and intracellular ADC concentration reaches an almost linear relationship (R^2^ ≥ 0.91) [12]. Regarding the internalization of the ADC-antigen complex, cleavable linker-based ADCs with membrane-permeable payloads seem to be less dependent on the trafficking of the antigen [10]. Such immunoconjugates are able to exert their cytotoxic activity after extracellular cleavage and subsequent local drug diffusion [13,14]. To this direction, several inert constituents of tumor microenvironment have been tested as stromal ADC-targets [15,16] and recently, a non-internalizing ADC was developed detaching its payload after consequent administration of a linker-activator, independently of endogenous cleavage [17]. Notably, this immunoconjugate was proved potent even against murine models insensitive to the FDA-approved ADC, BV. Minimal shedding should be also added in the list of beneficial antigen features, as secreted epitopes can bind to the circulated immunoconjugates and render them ineffective. This off-target antigen-ADC interaction may lessen the portion of the administered drug reaching into the tumor microenvironment and lead to unnecessary dose escalation [18]. The introduction of bispecific antibodies can help to overcome this technical issue [19]. However, in a modelling study, antigen shedding in solid malignancies may act positively by preventing binding site barrier (BSB) phenomenon and facilitating a more homogeneous distribution of ADC [20]. Therefore, these considerations about BSB phenomenon and bystander killing effect are challenging internalization and shedding as strict properties of ADC target [14].

Depending on the selected immunoglobulin subtype, the antibody component of ADC retains both targeted transport and cell-killing potential [21]. The main characteristics of candidate mAbs include high affinity for the target antigen, rapid internalization, favorable pharmacokinetic properties and minimal immunogenicity. The issue of immunogenicity has significantly improved with the introduction of human or humanized immunoglobulins [22]. Moreover, these antibodies interact better with both immune cells and complement system [23]. In ADC-engineering, most mAbs (chimeric, humanized or human) belong to IgG1 isotype while IgG3 isotype is not utilized [24]. The IgG3 isotype is cleared up to three times faster than IgG1, IgG2, and IgG4 (half-life of 7 days compared to 21 days) and is more sensitive to proteolysis, increasing the risk of immunogenicity [25,26]. The IgG1 isotype is usually employed because of its high affinity for all Fc-gamma receptors and its ability to induce secondary immune functions, antibody-dependent cellular cytotoxicity and complement-dependent toxicity [27]. On the other hand, IgG2 and IgG4 isotypes are poor inducers of the complement cascade [28]. The sub-nanomolar levels of mAbs prevent cross-reaction with off-target antigens, limit systemic toxicity and premature elimination [29], while the quick and tight binding to antigen enables rapid internalization and efficient payload delivery. However, similar to high antigen expression, high affinity can increase binding and rapid endocytosis of the prodrug within the first cancer cells, lowering the therapeutic efficacy of ADCs (BSB phenomenon) [30]. 

The bispecific ADCs (bsADCs) have been manufactured to identify a pair of different antigens or two distinct epitopes on the same antigen (known as biparatopic), conferring a more target-specific drug delivery compared to monospecific ADCs. A wide range of bispecific antibodies compositions have been described, with more than 100 formats reported in the literature [31,32]. Based on their robust selectivity, bsADCs improve the safety profile of conventional ADC formats and upgrade their applicability. Simultaneous engagement of co-expressed target antigens or non-overlapping epitopes allows an accumulated on-target toxicity, and diminishes uptake by adjacent healthy tissues [33]. Furthermore, bsADCs have been manipulated to enhance internalization and redirect lysosomal trafficking and degradation. The bsADCs can utilize strongly internalizing receptors to overcome suboptimal lysosomal uptake and limited drug exposure attributed to increased recycling of prone antigens such as HER2 [34]. Cross-linking between two molecules (e.g., a high-turnover membrane protein and a tumor-marker antigen), irrespective the affinity of mAb can accelerate subsequent downstream cascade. For instance, CD63 was proven to facilitate transmission from membrane to intracellular compartments. The identification of this high-yield molecular “shuttle” led to generation of anti-HER2/CD63, duostatin-3-linked bsADC. This bsADC displayed greater anti-tumor activity compared to monovalent HER2- and CD63 ADCs [35]. Prolactin receptor (PRLR) was pinpointed as another candidate target-antigen due to its rapid internalization and lysosomal delivery. The produced anti-HER2/PRLR bsADC not only boosts trafficking of HER2, but also displays greater activity than single HER2 ADC or PRLR ADC in breast cancer cells with intermediate HER2 and low PRLR levels [36]. Similarly, cross-linking of HER2 with the rapidly internalizing APLP2 receptor in DM1-linked bsADCs has also demonstrated promising findings in terms of potency, compared to single T-DM1 [37]. Another bsADC model was recently developed for CD7+/CD33+ acute myeloid leukemia (AML). Co-targeting of CD7 and CD33 increases the specificity of the immunoconjugate, compared to gemtuzumab ozogamicin (GO, approved anti-CD33 ADC), and augments cytotoxicity against CD7 + CD33+ cells both in vitro and in vivo [38]. Lastly, biparatopic platforms have been used to enhance specificity and maximize internalization and trafficking, as clustering and cross-linking of receptors accelerates intracellular trafficking. For this objective, Li et al. developed a biparatopicADC (MEDI4276), combining two non-overlapping HER2-targeted Abs, with activity across different levels of HER2 expression [39]. MEDI4276 is currently under investigation in a first-in-human clinical trial in patients with HER2+ breast or gastric cancer (NCT02576548) [40]. 

### 2.2. Linkers and Conjugation Technologies

Linkers have been designed to tether the cytotoxic molecule to the antibody scaffold, regulating several prodrug parameters such as circulation stability, solubility, and aggregation propensity. These components can generally be categorized into cleavable and non-cleavable ones, depending on whether they can be degraded or not. Cleavable linkers can be (i) acid-labile (e.g., hydrazones), (ii) reducible/glutathione-sensitive (e.g., disulfides), and (iii) protease-sensitive/peptide linkers. As an example of the first subgroup, Gemtuzumab ozogamicin (GO) employs a bifunctional 4-(4-acetylphenoxy) butanoic acid part attached to the calicheamicin payload via hydrazone linkage. This type of linker remains stable at normal blood pH but undergoes hydrolysis under lysosomal and endosomal acidic conditions [41] and possibly elsewhere in the body where pH is also low, resulting in undesired, nonspecific drug release. In 2010, this drawback led to the temporary withdrawal of GO, due to marked toxicity, attributable to linker instability [42]. In the second category, disulfide linkers exploit the transmembrane difference in reductive potential, owing to considerably higher intracellular concentrations of reducing agents [43]. Protease-sensitive/peptide linkers usually consist of oligopeptide substrates, most commonly dipeptide valine-citrulline (Val-Cit) combined with a self-immolative para- amino-benzyloxycarbonyl (PABC) spacer. This type of linker responds to overexpressed lysosomal proteases in cancer cells such as cathepsin B [44]. This last linker category combines well-established release patterns and improved drug control, and is already employed in approved ADC, BV [45,46]. Additional cleavable linker formats include β-glucuronide linkers, which are hydrophilic linkers responsive to β-glucuronidase, present in lysosomes and tumor necrotic areas. The hydrophilic composition of linker provides adequate polarity and stability and solubilizes typically hydrophobic payloads [47]. This masking through hydrophilic monodisperse polysarcosine drug-linker configuration (PSARlink) sensitive to β-glucuronidase, was applied in the production of novel high-DAR ADCs, against T-DM1-resistant cells [48]. Non-cleavable linkers display greater stability than cleavable ones, remaining intact through proteolytic, acidic and reductive conditions. ADCs with non-cleavable linkers depend their cytotoxic effects on the degradation of mAb scaffold. More specifically, the active metabolite is released into cytoplasm upon complete antibody breakdown leaving only a drug-linker-amino acid part [46,49]. Of note, amino acid capping increases hydrophilicity and reduces membrane permeability, influencing the bystander effect [50]. Examples of non-cleavable linkers include non-reducible thioether, as in T-DM1 [29]. Further advances on linker technology have been developed in order to optimize and expand ADCs’ utility. For instance, cleavable pyrophosphate-diester linkers in site-specific, glucocorticoid-bearing ADCs outside of oncological setting [51]; β-galactosidase-cleavable linkers for trastuzumab-MMAE conjugates [52] and dual enzyme-cleavable linkers (e.g., 3-*O*-sulfo-β-galactose linker), subjects of sequential cleavage by distinct lysosomal enzymes (e.g., arylsulfatase A and β-galactosidase) have demonstrated encouraging findings [53]. In the construction of dual-drug ADCs, flexible linkers contribute in the successful co-delivery of payloads with synergistic cytotoxic mechanisms [54]. Recently, Spangler et al. described a novel linker format, named as Fe(II)-reactive 1,2,4-trioxolane (TRX), which reacts with labile ferrous iron in cancerous tissue to induce a more tumor-selective drug release [55]. This TRX-linker limits the on-target-off-tumor toxicity upon uptake by adjacent healthy cells. A first-in-class platinum (II)-based metal-organic linker (Lx) was designed to surpass conventional linkage pitfalls, such as premature release. In preclinical studies, Lx-based ADCs have shown favorable safety profile and potency [56]. At the end, additional linkers including non-covalent DNA linkers (e.g., based on complementary oligonucleotide hybridization and base-pairing) [57] as well as photo-cleavable linkers on UV light-controlled ADCs [58], are also under clinical testing.

Conjugation affects the ADC stoichiometry and homogeneity, crosslinking the cytotoxic drug-linker moiety to the antibody vehicle. The conjugation strategy dictates the quantity of drug molecules attached per antibody, defined as drug-to-antibody ratio or DAR. Of note, broader DAR distribution produced more heterogenous ADC, which results in product inconsistency and suboptimal efficiency [46]. Conventional conjugation techniques utilize intrinsically nucleophilic side-chain groups of solvent-accessible amino-acid residues in the mAb component, with native lysine and cysteine residues being the most frequently detected. Utilizing native lysine residues can lead to highly heterogenous ADC species because of their relative abundance (>80) in a typical IgG molecule and the wide range of possible conjugation spots. Regarding native cysteines, in IgG1 they form 16 pairs; 4 interchain and 12 intrachain disulfide bridges. Cysteine-based conjugation is based on reduction of the interchain cysteines and can spawn up to eight sulfhydryl(-SH) groups, thus yielding DARs ≤ 8. The nucleophilic sulfhydryl groups consequently can be reacted with electrophilic entities to allow conjugation through various chemical reactions [45,49]. Recently, efforts have been concentrated on more homogenous drug loading and better-controlled DARs. Disulfide re-bridging can be applied without requiring recombinant or enzymatic modifications [59]. Selective mutations of the mAb amino-acid sequence enable site-specific conjugation via recombinant incorporation of reactive handles. THIOMAB™ technology introduces two genetically engineered, unpaired cysteines, spares interchain disulfides, and permits selective attachment. In vivo studies have shown improved therapeutic window and tolerability of developed ADCs with sustained DAR ~ 2 and high homogeneity (>90%) [60]. Another site-specific conjugation approach is based on the introduction of unnatural amino-acids (uAA) with reactive side-chains for chemical tethering. Installation of the non-canonical residues is feasible through recombinant technology [61,62]. Successful enzymatic ligation techniques have also been used for site-selective bioconjugation of native or engineered mAbs with attractive partners. Microbial transglutaminase (mTG) catalyzes the formation of isopeptide bond between linker and glutamine in de-glycosylated mAbs, without modifying native glutamines [63,64]. In addition, short glutamine motif (LLQG) can be inserted into mAbs, rendering them fit for peptide sequence-specific linking via transpeptidation in presence of mTG. This technique generates ADCs with strictly controlled DARs and favorable profiles compared to traditional ADCs [65]. Interestingly, mTG-mediated ligation has also been used for branched linkers in order to flexibly increase payload loading of ADCs, without further intervening in the mAb structure to accommodate multiple individual linkers [66]. Moreover, microbial sortase A (SortA) can recognize a specific pentapeptide tag (LPETG) appended to C-terminus of recombinant mAbs and mediate the conjugation. This sortase-mediated antibody conjugation technology (SMAC^TM^) can be used to efficiently yield site-specific homogenous ADCs with predefined DARs that retain the tumor killing properties of traditional ADCs [67]. SmartTag^TM^ (Specific Modifiable Aldehyde Recombinant Tag) is another enzyme-assisted platform that utilizes a formylglycine-generating enzyme (FGE) to recognize a CxPxR (X: serine, threonine, alanine, or glycine) tag and convert cysteine to formylglycine residues bearing a reactive aldehyde group. Localized bioconjugation is achieved by modifying the mAb and selectively inserting the FGE-recognized sequences at intended sites [68]. Other remodeling techniques are focused on mAbs’ glycosylation. These strategies can modify the conserved N-glycan chain of Fc domain to allow conjugation [69], or can incorporate reaction handles via placement of non-natural saccharides [70].

### 2.3. Payloads

In cancer settings, the employed warhead is a super-toxic compound potent in sub-nanomolar concentrations and, thus, intolerable if administered unconjugated. The ADCs’ payloads are categorized into two groups: DNA-damaging agents and microtubule-disrupting agents. DNA-damaging drugs are further divided into three subcategories: DNA-double strand break inducing agents, DNA alkylators, and DNA intercalators. Microtubule-disrupting agents are the most widely used cytotoxic agents in ADC technology. Maytansinoids and auristatins are the main representatives. A plethora of other drug classes targeting other cellular processes are investigated as potential ADC payloads. The payload classes of FDA-approved ADCs as well as the attached drugs for non-oncologic ADCs are discussed below. 

#### 2.3.1. Calicheamicins

Calicheamicins are a class of natural anticancer antibiotics isolated from the actinomycete *Micromonospora echinospora* spp. *Calichensis* [71]. These compounds exhibit site-specific binding in the minor groove of DNA. The primary recognition site is TCCT/AGGA. Subsequently, reductive cleavage by cellular thiols generates a diradical species that abstracts hydrogen atoms from DNA inducing strand scission and cell death [72]. A semisynthetic derivative of chalicheamicin, N-acetyl-gamma calicheamicin 1,2-dimethyl hydrazine, is already used in two FDA-approved ADCs, inotuzumab ozogamicin and gemtuzumab ozogamicin. Although potent, these ADCs have certain limitations such as increased aggregation and shortened half-life, due to the employed linker chemistry. Next-generation calicheamichin-based ADCs exhibit these features to a lesser degree due to novel site-specific conjugation [73].

#### 2.3.2. Pyrrolobenzodiazepines (PBDs)

Pyrrolobenzodiazepines (PBDs) bind on sequence-specific fragments of opposite DNA strands and stop their separation during the cell cycle (e.g., G2/M boundary), inducing cell death [74]. Importantly, PBD dimers can permeate membranes and have a very short half-life. Therefore, these drugs exert bystander killing activity with limited systemic accumulation. Furthermore, they exhibit small tendency for acquired resistance and technically reversible alkylation after DNA digestion and heating [75,76]. The latter characteristic enables the quantitation of PBD compound and isolated DNA, comparing payload exposure in tumor and normal tissue, by the levels of calculated alkylation [62]. This ratio accurately represents the safety and efficacy profile of PBD-containing ADC. The latest FDA-approved ADC, loncastuximab tesirine, employs tesirine as its PBD warhead [77].

#### 2.3.3. Auristatins

Auristatins comprise synthetic analogues of the natural cytotoxic product Dolostatin 10, isolated from *Dolabella auricularia.* These drugs block mitosis via inhibition of tubulin polymerization, and thus lead to apoptosis [78]. Two synthetic auristatin derivatives, monomethyl auristatin E (MMAE) and monomethyl auristatin F (MMAF), have been studied in great detail. MMAF is less effective compared to MMAE because of the charged phenylalanine in its C-terminal that presumably hinders intracellular access [79]. However, MMAF is highly potent once it has reached its target being one of the most toxic auristatins generated. In contrast to MMAE, MMAF does not exert bystander killing activity. A novel auristatin payload, auristatin F-hydroxypropylamide (AF-HPA), has been recently reported in the context of the pioneering ADC platform [80]. Interestingly, the membrane permeating AF-HPA undergoes intracellular conversion to membrane non-permeating MMAF, resulting in a controlled bystander effect. This characteristic decreases the rate of neutropenia as a dose-limiting toxicity of these ADCs compared to other auristatin platforms [81]. Four FDA-approved ADCs contain auristatins as their attached payload. 

#### 2.3.4. Maytansinoids

Maytansinoids are derivatives of maytansine which is a benzoansamacrolide isolated from *Maytenus ovatus.* Maytansinoids act as antimitotic agents, binding to tubulin at or near the vinca binding site. Tubulin binding destabilizes the microtubule assembly and induces cell cycle arrest at the G2/M phase [2]. Maytansinoids are hydrophobic and upon extracellular or intracellular cleavage they can effectively diffuse into antigen-negative cells. Trastuzumab emtasine (T-DM1) utilizes DM1, a thiol-containing maytansine derivative, as its warhead. 

#### 2.3.5. Camptothecin (CPT)

Camptothecin (CPT) is pentacyclic quinoline alkaloid originally isolated from the plant species *Camptotheca acuminata.* CPT and its analogues inhibit topoisomerase-1 activity. CPT interacts with topoisomerase-1 and DNA interface through hydrogen bonding, and form reversible complexes, blocking DNA replication and inducing cell death [82]. SN-38, an active metabolite of irinotecan, and exatecan, a water-soluble CPT derivative, are the payloads of FDA-approved sacituzumab govitecan and trastuzumab deruxtecan, respectively. 

#### 2.3.6. Dual-Drug ADCs

Dual-drug ADCs co-deliver different payloads with complementary or synergistic cytotoxic mechanisms at equimolar concentrations [83]. This co-administration of anti-cancer agents with multi-loading linkers and dual payloads can counteract tumor resistance. Data have shown that cancer cells, resistant to an ADC, remain sensitive to the alternate payload delivered via the same mAb [84]. Levengood et al. reported an homogeneous dual-auristatin ADC carrying two tubulin polymerization inhibitors, MMAE and MMAF [85]. This novel ADC was potent against xenograft models of anaplastic large cell lymphoma refractory to monotherapy with either of the individual drugs, further supporting the development of this concept. To this end, another dual-drug ADC carrying two mechanistically different warheads was subsequently described. This approach combined MMAE and a PBD-dimer and exerted two distinct cytotoxic mechanisms consistent with the tethered payloads [54]. However, this PBD-dimer/MMAE-ADC failed to achieve enhanced cell-killing efficacy compared to the ADC equipped with the PBD-dimer alone, probably because of the great potency of the latter payload. A comparable ADC design carrying a DNA alkylator and a microtubule inhibitor was published by Duvall et al. [86]. Similar to Kumar et al. [54], Nilchan et al. developed a dual-drug ADC (PNU-159682/MMAF) against HER2(+) cell lines that did not exhibit greater potency compared to single-PNU-159682 ADC [87]. MMAE and MMAF were also combined in a dual-drug ADC format for HER2(+) breast cancer lines [88]. Interestingly, this construct was more efficient than both monotherapy with single-drug ADCs and administration of the two variants, as MMAF-induced cell death of HER2(+) cells enabled MMAE bystander killing activity. 

## 3. Bystander Killing and Resistance Phenomena

### 3.1. Bystander Killing Effect

The bystander killing effect is exhibited when the released cytotoxic drug of ADC is unleashed into surrounding antigen-negative tumor and/or normal cells. The naked payload, in cleavable linkers, or more rarely, the drug-linker-amino acid fraction in non-cleavable linkers, can diffuse through the phospholipid bilayer towards the nearby cells [89]. A critical parameter affecting the extent of the phenomenon is the membrane permeability of the bioactive form of the drug. Charged and hydrophilic ADC drug derivatives are known to demonstrate a minimal bystander effect, while more hydrophobic and neutrally charged payload catabolites experience the effect to a greater degree [50,90]. The bystander activity can be quantified in vitro by incubating the ADC agent with co-cultures of cancer cells with a certain number of antigen negative cells and varying numbers of antigen positive cells. The cytotoxic potency of tethered payload is determined by the number of antigen positive cells required to kill the antigen negatives [91,92]. In an attempt to further quantify and predict the clinical impact of the bystander effect, several pharmacokinetic and pharmacodynamic modelling approaches have been recently proposed [93,94,95], but this issue remains under debate. To a certain degree, the bystander killing effect can be exploited to tackle tumor heterogeneity. In this case, the effect of the ADC-attached drug depends less on the homogenous expression of the target antigen, because its surface expression on all tumor cells is not required [96]. The FDA-approved ADC, Trastuzumab deruxtecan, serves as a great example of how the bystander killing mechanism can be translated into therapeutic benefit [97]. More specifically, in vivo and in vitro preclinical testing in cell lines with varying HER2 expression profile revealed notable antineoplasmatic activity against every antigen expression density [98]. On the other side, the bystander effect when experienced on normal neighboring cells can contribute to undesirable off-target toxicity, hampering the safety profile of administered immunoconjugate. Modern auristatin platforms, such as upifitamab rilsodotin (XMT-1536), with low bystander killing effect are causing less neutropenia compared to traditional auristatin-containing ADC [81]. It is worth-noting that the bystander effect was the functional basis for the development of non-internalizing ADCs [14].

### 3.2. Drug Resistance

Similar to other anti-cancer drugs, many different resistance mechanisms have been developed by tumor cells to overcome ADC-based treatments and are listed below.

#### 3.2.1. Antigen-Related Resistance

Downregulation of antigen expression is a major mechanism of drug resistance that usually develops in cells chronically exposed to the ligand [99]. Breast cancer cell lines, 361-TM and JIMT1-TM, exposed to multiple cycles of an anti-HER2 trastuzumab–maytansinoid ADC (TM-ADC) expressed 25% and 58% reduction of binding ability, respectively [84]. Masking of the targeted epitope contributes to additional antigen-related resistance [100].

#### 3.2.2. Deficient Lysosomal Function

Impaired lysosomal proteolytic activity delays ADC degradation, limiting its cell-killing efficacy. In T-DM1 insensitive clones, although no alterations were detected in endocytosis and intracellular trafficking, the proteolytic activity within the lysosomes was deficient due to the increased pH [101]. Aberrant vacuolar H^+^-ATPase (V-ATPase) activity has been observed in resistant cells, while administration of the V-ATPase inhibitor, bafilomycin A1, successfully sensitize again cell lines [102]. Another report proposed the use of photoactivatable nanoparticles to manipulate lysosomal pH levels and restore the anti-tumoral activity of ADCs [103]. At this level, cells’ resistance can also be induced by disruption of transportation through the lysosomal membrane to the cytosol [104]. Especially in cases of non-cleavable linked ADCs, silencing of lysosomal transporters can cause intralysosomal accumulation of the non-cleavable catabolite, decreasing ADC activity [105].

#### 3.2.3. Upregulated Efflux Pumps

The efflux of bioactive payload via ATP binding cassette (ABC) transporters, such as MDR1/PgP, is another mechanism of acquired resistance. Notably, the majority of commonly used ADC payloads are substrates of efflux transporters [100]. Indeed, Chen et al. observed that MMAE, employed by BV, was actively exported in HL cells resistant to BV [106]. Subsequent in vivo studies proved that PgP inhibition could restore sensitivity to BV in BV-resistant HL cell lines [107]. In a phase I clinical trial the co-administration of BV with cyclosporine A (MDR-modulator) achieved responses even in BV-refractory HL patients [107]. In another example, Loganzo et al. detected increased ABCC1 (MRP1) as possible resistance mediator in 361-TM cells and intriguingly showed that switching a non-cleavable linker for a cleavable one could restore sensitivity to TM-ADC [84]. 

#### 3.2.4. Survival/Apoptotic Signaling

Alterations in survival signaling pathways can also modulate crucially ADC cytotoxicity [99,100,104]. Elevated PI3K/AKT/mTOR pathway activity has been associated with acquired resistance to GO in AML cells [108] via overproduction of anti-apoptotic factors [99]. PI3K/AKT/mTOR pathway inhibition via MK-2206, an AKT inhibitor, or via PP242, a mTOR1/2 inhibitor, resulted in re-sensitization of resistant cells to GO [108,109]. Furthermore, depletion of PTEN expression, a negative PI3K pathway regulator and aberrant activation of STAT3 were also correlated with resistance to T-DM1 [110,111]. Lastly, upregulation of anti-apoptotic proteins BCL-2/BCL-X induces important resistance [112] while their inhibition was shown to restore sensitivity to T-DM1 [113].

#### 3.2.5. Binding-Site Barrier (BSB) Phenomenon

The binding-site barrier (BSB) phenomenon is defined as the binding-dependent penetration and non-uniform distribution of the ADC into the tumor microenvironment [114]. The extensive cellular uptake in the perivascular/peritumoral regions reduces the penetration and homogeneous diffusion into the main mass [115], affecting the cytotoxic effect of particular ADC that do not exhibit effective bystander killing activity [116]. Antigen shedding [20], sizing and affinity alterations [117,118], co-administration of molecules interfering with binding [119] and, more recently, dosing modifications [120] have been described as strategies to attenuate the clinical impact of BSB. Cilliers et al. reported that co-administration of trastuzumab emtasine (T-DM1) with trastuzumab effectively modifies distribution of T-DM1 in HER2(+) NCI-N87 tumor cells. This approach was tested using two different trastuzumab-based ADCs exhibiting different bystander activity, at two different doses and in two different HER2(+) cancer models [114]. Co-administration approach was more efficient in the case of ADC without bystander killing ability in tumor models with very high antigen surface density. In gynecological malignancies, higher doses of mesothelin-targeting ADC in longer intervals were reported to produce better outcomes, overcoming BSB, compared to lower doses administered more frequently [120]. 

## 4. Applications of ADCs for Oncological and Non-Oncological Conditions

As previously discussed in detail, ADCs have been incorporated in oncological therapeutic algorithms and have shifted treatment landscape from conventional chemotherapy to the era of molecularly targeted medicine. In just the last two years, six novel ADC agents have been approved by FDA for anticancer indications. Table 1 summarizes all these approved oncological ADCs, presenting their main characteristics. The preclinical and clinical data that drove these ADCs to their regulatory approvals have been published recently by our team [1]. In addition to these indications, many other solid malignancies, including prostate cancer [121], gastric cancer [122], pancreatic cancer [123], and hepatocellular carcinoma [124] have been entered into the focus. Table 2 presents some late-stage trials on currently non-approved oncological ADCs.

Being on preliminary steps, ADC technology is gradually tested into a broader spectrum of diseases beyond the sphere of oncology. For such non-oncological implications, the types of payloads vary from glucocorticoid receptor modulators and kinase inhibitors to antibiotics and siRNA (Table 3). Notable innovative approaches have produced anti-inflammatory ADCs binding dexamethasone or other immunomodulatory drugs to mAbs. In 2012, Graversen et al. reported the development of a biodegradable anti-CD163 dexamethasone conjugate that selectively delivers the glucocorticoid to macrophages [125]. Measuring in vitro the suppression of TNF-a secretion by rat macrophages, the ADC demonstrated approximately 50-fold higher anti-inflammatory activity compared to the unconjugated dexamethasone. The in vitro efficacy was replicated by in vivo findings and the anti-inflammatory activity of this ADC was consistently confirmed by several other preclinical studies [126,127]. 

ABBV-3373 is a novel antibody-glucocorticoid conjugate that is currently being tested for the treatment of moderate/severe rheumatoid arthritis. ABBV-3373 was constructed by conjugating a glucocorticoid receptor mediator to an anti-TNFa mAb and qualified to clinical evaluation after displaying significant properties in mouse models of arthritis [5]. In a phase II trial (NCT03823391) completed in August 2020, 48 patients were randomly allocated in a 2:1 ratio to receive ABBV-3373 or adalimumab [128]. The primary endpoint, change in Disease Activity Score 28 C-Reactive Protein, was significantly greater in the experimental arm compared to the control arm (−2.65 versus −2.13, respectively, *p* = 0.022). In terms of safety, the adverse events (AEs) rate was lower in the ADC group (35% vs 71%, respectively).

In the context of infectious diseases, DSTA4637S is the first attempt to develop an antibody-antibiotic conjugate (AAC). DSTA4637S is designed to target and eliminate intracellular reservoirs of *Staphylococcus aureus*. DSTA4637S comprises an IgG1 mAb (MSTA3852A) against a *S. aureus* antigen, β-N-acetylglucosamine cell-wall teichoic acid (β-GlcNAc-WTA), conjugated to a rifamycin derivative, 4-dimethylamino piperidino-hydroxybenzoxazino rifamycin (dmDNA31), via a val-cit linker [129]. Its action is based on the opsonization of bacteria and the subsequent endocytosis by the host macrophages. Upon internalization, phagolysosomal process results in linker cleavage and antibiotic release. Once dmDNA31 is liberated, it kills AAC-opsonized and pre-existing bacteria within the phagocytes. DSTA4637S remains stable in circulation after intravenous administration, with minimal antibiotic deconjugation reported [130]. In mouse models, the pharmacokinetic profile was similar between *S. aureus* infected and non-infected subjects [131]. Notably, a single dose of the AAC resulted in a significant reduction of bacterial load in infected mice for 14 days following the administration. This prolonged bactericidal efficiency is attributed to the extended half-life of the antibiotic once conjugated (unconjugated vs. conjugated half-life: 3–4 h versus 4 days) [131]. In a subsequent study, whole-body bioluminescence imaging was used to examine the antibacterial activity of the conjugate as a monotherapy and in combination with vancomycin in mice injected with luminescent *S. aureus.* AAC administration yielded persistent bioluminescent intensity reduction, while it also achieved augmented potency in the vancomycin combinatorial regimen [132]. Based on these data, the pharmacokinetic and safety profiles of DSTA4637S were investigated in healthy volunteers in a phase I trial (NCT02596399). No subject withdrawals and no serious AEs were reported upon study completion [6]. Another phase I study (NCT03162250) examining safety and tolerability of DSTA4637S in patients with MRSA (Methicillin-resistant *Staphylococcus aureus*) and MSSA (Methicillin-susceptible *Staphylococcus aureus*) bacteremia was recently completed and its results are expected.

Among FDA-approved ADCs, BV is the only agent currently evaluated for a non-oncological condition. BV involves a chimeric anti-CD30 IgG1 tethered via a protease-cleavable linker to the MMAE, bearing DAR = 4 [133]. The efficacy of BV in the treatment of diffuse cutaneous systemic sclerosis is currently tested in two phase II trials (NCT03222492, NCT03198689). According to an interim report of second study, BV has already met the primary endpoint at 24 weeks after treatment initiation (decrease in modified Rodnan skin score of ≥8) [4]. BV has been previously investigated for steroid refractory acute graft versus host disease (NCT01616680) and systemic lupus erythematosus (NCT02533570); however, both trials were discontinued.

**Table 3:** Summary of ADCs tested for non-oncological indications.

ADC	Indication	Antibody	Linker	DAR	Testing Status	Initial Publication, Year	Reference
Anti-E Selectin dexamethasone (Dexa–AbhEsel)	Chronic models of inflammation	Murine anti-E-selectin mAb (H18/7)	Succinate linker	2.3	In vitro preclinical	J Immunol, 2002	[134]
Anti-CD163 dexamethasone (*Cymac-001)*	Chronic models of inflammation	Murine anti-CD163 mAb (Ed-2)	Hemisuccinate linker	~4	In vivo preclinical	Mol Ther, 2012	[125]
Anti-CD74 fluticasone propionate (Anti-CD74-flu449)	Autoimmune models	Human anti-CD74 mAb	Pyrophosphate acetal linker	≥1.7	In vivo preclinical	Bioconjug Chem, 2018	[135]
Anti-CD70 budesonide	Chronic models of inflammation	Murine anti- CD70 mAb (Bu69)	CatPhos linker	1.9	In vitro preclinical	Bioconjug Chem, 2016	[136]
Anti-CXCR4 dasatinib	Autoimmune and inflammatory models	Humanized anti-CXCR4 mAb (HLCX)	Tetra-poly-ethylene glycol linker	~3	In vitro preclinical	J Am Chem Soc, 2015	[137]
Anti-CD11a PDE4 inhibitor	Chronic models of inflammation	Humanized anti-CD11 mAb	PEG4-Phe-Lys	~2	In vivo preclinical	Mol Ther, 2016	[138]
Anti-CD11a LXR agonist	Atherosclerosis	Humanized anti-CD11 mAb	PEG4-Phe-Lys	2	In vitro preclinical	Bioconjug Chem, 2015	[139]
Anti-CD71 siRNA	Muscular diseases	Murine ant-CD71 mAb	Maleimide linker	N/A	In vivo preclinical	J Control Release, 2016	[140]
Anti-TNFRSF13c siRNA	Myasthenia gravis	Murine anti-TNFRSF13c mAb	Protamine linker	N/A	In vivo preclinical	Clin Immunol, 2017	[141]
Anti-IL-7R MMAE (A7R-ADC-MMAE)	Steroid-resistant arthritis	Murine anti-IL-7R mAb	Val-Cit linker	N/A	In vivo preclinical	Sci Rep, 2017	[142]
Anti-CD30 vedotin (ADCETRIS)	Systemic sclerosis	Chimeric anti-CD30 mAb (cAC10, SGN-30)	Val-Cit linker	~4	Phase II clinical trial (NCT03198689), (NCT03222492)	Ann Rheum Dis, 2021	[4]
Anti-CD117 saporin (C117-ADC)	Conditioning for HSCT	Murine anti-CD117 mAb	N/A	N/A	In vivo preclinical	Nat Commun, 2019	[143]
Anti-CD45 saporin (CD45-SAP)	Conditioning for HSCT	Murine anti-CD45 mAb	N/A	N/A	In vivo preclinical	Nat Biotechnol, 2016	[144]
Anti-IL-6 alendronate	Rheumatoid arthritis	Humanized anti-IL-6 mAb (tocilizumab)	PDPH-PEG-NHS	N/A	In vivo preclinical	Bioconjug Chem, 2017	[145]
Anti–C5aR1 C5 siRNA	Rheumatoid arthritis	Murine anti-C5aR1 mAb	Protamine linker	N/A	In vivo preclinical	J Immunol, 2015	[146]
Anti-FRβ Pseudomonas exotoxin A (PE38)	Rheumatoid arthritis	Murine anti-FRβ mAb	N/A	N/A	In vivo preclinical	Arthritis Rheumatol, 2006	[147]
Anti-TNFα glucocorticoid (ABBV-3373)	Rheumatoid arthritis	N/A	N/A	N/A	Phase II clinical trial (NCT03823391)	Ann Rheum Dis, 2021	[5]
Anti-*S. aureus* antibiotic (DSTA4637S)	*S. aureus* bacteremia	Human anti-β-N- acetylglucosamine cell-wall teichoic acid (β-GlcNAc- WTA) mAb	MC-Val-Cit-PAB-OH	2	Phase I clinical trial (NCT03162250)	Nature, 2015	[129]

Abbreviations: DAR: Drug-to-Antibody Ratio; mAb: monoclonal antibody N/A: not available.

## 5. Conclusions

Capitalizing on the extensive research of last decade, ADCs are entering into a phase of exponential growth. Accumulating clinical and preclinical experience will guide the production of agents with greater potency and better therapeutic window than parental compounds. In the oncological setting, promising strategies such as bispecific antibodies and dual-drug ADCs are expected to overcome limitations of first-generation ADCs. At the same time, the preliminary implications of ADC pioneering technology outside of the oncological sphere are expected to extend this tempered optimism in a variety of other non-oncological diseases.

## Figures and Tables

**Table 1:** Current indications and characteristics of FDA-approved ADCs.

ADC	Manufacturer	Year of Initial FDA Approval	Indications	Target	Antibody	Payload	Linker	DAR	Common Adverse Events (>10%)
**Gemtuzumab Ozogamicin** **(Mylotarg^®^, CMA** **-676)**	Pfizer/Wyeth	2000, withdrawn 2010, re-approved 2017	Newly diagnosed (de novo) CD33+ AML in adults (as a monotherapy or combined with chemotherapy) and pediatric patients 1 month and older (combined with chemotherapy) and relapsed/refractory CD33+ AML in adults and pediatric patients ≥ 2 years of age	CD33	Humanized IgG4	Calicheamicin derivative	Acid-labile hydrazone linker	~2–3	Infection, hemorrhage, thrombocytopenia, hypophosphatemia, hypokalemia, hyponatremia, nausea, vomiting, elevated ALP, elevated aminotransferase, fatigue, febrile neutropenia, constipation, abdominal pain, pyrexia, mucositis
**Brentuximab Vedotin** **(Adcetris^®^, SGN-35)**	Seattle Genetics, Millennium/ Takeda	2011	Previously untreated Stage III/IV cHL (combined with chemotherapy), cHL at high risk of relapse or progression as post-auto-HSCT consolidation, cHL after failure of auto-HSCT or after failure of ≥ 2 prior chemotherapy regimens, previously untreated sALCL or other CD30+ peripheral T-cell lymphomas (combined with chemotherapy), relapsed sALCL, relapsed peripheral cutaneous ALCL or CD30+ MF	CD30	Chimeric IgG1	MMAE	Protease-cleavable dipeptide (Val-Cit) linker	~4	Neutropenia, peripheral sensory neuropathy, fatigue, upper respiratory tract infection, nausea, diarrhea, anemia, thrombocytopenia, pyrexia, rash, abdominal pain, vomiting, arthralgia, myalgia, pruritus, peripheral motor neuropathy, headache, constipation, dizziness, lymphadenopathy, dyspnea, back pain, anxiety
**Ado-trastuzumab emtansine ** **(T-DM1, Kadcyla^®^)**	Genentech, Roche	2013	Unresectable locally advanced or metastatic HER2+ breast cancer, previously treated with trastuzumab and a taxane, adjuvant treatment for HER2+ early breast cancer with residual invasive disease after neoadjuvant taxane and trastuzumab	HER2/ERB2	Humanized IgG1	DM1	Thioether (non-cleavable) linker	3.5	Nausea, constipation, diarrhea, vomiting, abdominal pain, dry mouth, stomatitis, headache, peripheral neuropathy, dizziness, epistaxis, cough, dyspnea, fatigue, musculoskeletal pain, arthralgia, myalgia, pyrexia, thrombocytopenia, anemia, increased aminotransferases, insomnia, rash, hypokalemia
**Inotuzumab ozogamicin** **(Besponsa^®^, CMC-544)**	Pfizer/Wyeth	2017	Relapsed or refractory CD22+ B-cell precursor ALL in adults	CD22	Humanized IgG4	Calicheamicin derivative	Acid-labile hydrazone linker	~4	Thrombocytopenia, neutropenia, infection, anemia, leukopenia, nausea, fatigue, hemorrhage, pyrexia, elevated transaminases, febrile neutropenia, elevated gamma-glutamyltransferase, lymphopenia, headache, abdominal pain, diarrhea, constipation, vomiting, stomatitis, elevated ALP
**Polatuzumab vedotin-piiq** **(Polivy^®^, DCDS4501A, RG7596)**	Genentech, Roche	2019	Relapsed or refractory diffuse large B-cell lymphoma (combined with bendamustine and rituximab) in adult patients after ≥ 2 prior therapies	CD79b	Humanized IgG1	MMAE	Protease-cleavable dipeptide (Val-Cit) linker	3.5	Neutropenia, thrombocytopenia, anemia, leukopenia, lymphopenia, febrile neutropenia, peripheral neuropathy, dizziness, diarrhea, vomiting, infusion-related reactions, pyrexia, decreased appetite, fatigue, pneumonia, upper respiratory tract infection, decreased weight, hypokalemia, hypoalbuminemia, hypocalcemia
**Enfortumab vedotin** **(Padcev^®^, AGS-22M6E, AGS-22CE)**	Astellas/ Seattle Genetics	2019	Locally advanced or metastatic urothelial cancer in adult patients who had received prior treatment with a PD-1/L1 inhibitor and platinum-based chemotherapy in neoadjuvant/adjuvant setting	Nectin 4	Fully human IgG1	MMAE	Protease-cleavable dipeptide (Val-Cit) linker	~3.8	Peripheral neuropathy, dysgeusia, fatigue, decreased appetite, rash, alopecia, dry skin, pruritus, dry eye, nausea, vomiting, constipation
**Fam-trastuzumab deruxtecan-nxki ** **(Enhertu^®^, DS-8201a, T-DXd)**	AstraZeneca/Daiichi Sankyo	2019	Unresectable or metastatic HER2+ breast cancer in adult patients who have previously received ≥ 2 HER2 blockade regimens in the metastatic setting, locally advanced or metastatic HER2+ gastric or gastroesophageal adenocarcinoma after trastuzumab-based treatment	HER2/ERB2	Humanized IgG1	DXd (exatecan derivative)	Protease-cleavable tetrapeptide (Gly-Gly-Phe-Gly) linker	7–8	Nausea, vomiting, constipation, diarrhea, abdominal pain, stomatitis, dyspepsia, fatigue, alopecia, rash, decreased appetite, hypokalemia, anemia, neutropenia, leukopenia, thrombocytopenia, cough, dyspnea, epistaxis, headache, dizziness, upper respiratory tract infection, dry eye
**Sacituzumab govitecan-hziy ** **(Trodelvy^®^ IMMU-132, HRS7-SN38)**	Immunomedics	2020	Unresectable locally advanced or metastatic triple negative (HR-/HER2-) breast cancer after ≥2 prior systemic therapies, locally advanced or metastatic urothelial carcinoma after platinum-based chemotherapy and either a PD-1 or PD-L1 inhibitor	Trop-2	Humanized IgG1	SN-38	HydrolysableCL2A linker	7.6	Nausea, diarrhea, neutropenia, fatigue, anemia, vomiting, constipation, alopecia, rash, headache, respiratory tract infection, decreased appetite, urinary tract infection, hyperglycemia, arthralgia, dyspnea, dizziness, neuropathy, back pain, edema, thrombocytopenia, hypomagnesemia, hypokalemia, hypophosphatemia, pruritus, mucositis
**Belantamab mafadotin-blm ** **(Blenrep^®^, GSK2857916)**	GlaxoSmithKline	2020	Relapsed or refractory multiple myeloma in adult patients who have received ≥ 4 therapies, including an anti-CD38 mAb, a proteasome inhibitor and an immunomodulatory agent	BCMA	Afucosylated Humanized IgG1	MMAF	Maleimidocaproyl (mc) linker	~4	Keratopathy, decreased visual acuity, blurred vision, dry eyes, nausea, diarrhea, constipation, blurred vision, pyrexia, infusion-related reactions, arthralgia, back pain, upper respiratory tract infections, decreased appetite, fatigue
**Loncastuximab tesirine-lpyl ** **(Zynlonta^®^, ADCT-402)**	ADC Therapeutics	2021	Relapsed or refractory large B-cell lymphoma in adult patients after ≥ 2 lines of systemic therapy	CD19	Humanized IgG1	PDB dimer SCX (SG3199)	Protease-cleavable valine-alanine linker	2.8	Fatigue, edema, rash, pruritus, nausea, diarrea, abdominal pain, vomiting, constipation, musculoskeletal pain, decreased appetite, dyspnea, pleural effusion, upper respiratory tract infection

Abbreviations: DAR: drug-to-antibody ratio; hSCT: Hematopoietic Stem Cell Transplant; MMAE: Monomethyl Auristatin E; MMAF: Monomethyl Auristatin F; DM1: maytansine 1 derivative; HER2: human epidermal growth factor receptor 2; ALP: alkaline phosphatase; AML: acute myeloid leukemia; cHL: classic Hodgkin lymphoma; sALCL: systemic anaplastic large cell lymphoma; MF: mycosis fungoides; ALL: acute lymphoblastic lymphoma; HR: hormonal receptor; mAB: monoclonal antibody; PBD: pyrrolobenzodiazepine.

**Table 2:** Summary of non-approved ADCs that have been granted breakthrough therapy designation, fast-track designation or priority review by the US FDA and their current clinical trial status.

ADC Name	FDA Status	Target/Payload	NCT Number	Current Trial Status	Indication	Assigned Interventions
Tisotumab vedotin (TF-011-MMAE)	*Priority review granted in April 2021*	Tissue factor/MMAE	NCT04697628 (innovaTV 301)	3	Second or Third-line Recurrent or Metastatic Cervical cancer	Tisotumab vedotin 2.0 mg/kg IV Q3W Topotecan 1 or 1.25 mg/m2 IV on D1-5 Q3W Vinorelbine 30 mg/m2 IV on D1 and 8 Q3W Gemcitabine 1000 mg/m2 IV on D1 and 8 Q3W Irinotecan 100 or 125 mg/m2 IV weekly for 28 days, Q6W Pemetrexed 500 mg/m2 IV on D1 Q3W
Trastuzumab duocarmazine (SYD985)	*Fast Track designation granted in January 2018*	*HER2/seco*-*DUBA*	NCT03262935 (TULIP)	3	HER2+ unresectable locally advanced or metastatic breast cancer	Trastuzumab duocarmazine RP2D 1.2 mg/kg IV Q3W Lapatinib + capecitabine or Trastuzumab + capecitabine or Trastuzumab + vinorelbine Trastuzumab + eribulin
Mirvetuximab soravtansine (IMGN853)	Fast Track designation granted in June 2018. Accelerated approval pathway includes pivotal trial SORAYA and confirmatory trial MIRASOL	Folate receptor α/DM4	NCT04296890 (SORAYA)	3	Platinum-resistant advanced high-grade epithelial ovarian, primary peritoneal, or fallopian tube cancer, with high Folate receptor α expression	Mirvetuximab soravtasine 6 mg/kg IV Q3W
			NCT04209855 (MIRASOL)	3	Platinum-resistant advanced high-grade epithelial ovarian, primary peritoneal or fallopian tube cancer with high Folate receptor α expression	Mirvetuximab soravtasine 6 mg/kg IV Q3W Paclitaxel 80 mg/m^2 QW within a 4-week cycle or Pegylated liposomal doxorubicin 40 mg/m^2 Q4W or Topotexan 4 mg/m^2 IV either on D1, 8, 15 Q4W or 1.25 mg/m^2 on D1-5 Q3W
Upifitamab rilsodotin (XMT-1536)	Fast Track designation granted in August 2020	NaPi2b/DolaLock (auristatin F- hydroxypropylamide payload molecules)	NCT03319628 (UPLIFT; Pivotal Cohort)	1b/2	Platinum-resistant ovarian cancer and non-small cell lung cancer, adenocarcinoma subtype	Upifitamab rilsodotin RP2D IV Q4W
Disitamab vedotin (RC48)	Breakthrough Therapy designation granted in September 2020	HER2/MMAE	NCT04879329	2	HER2+ locally advanced or metastatic urothelial carcinoma in second-line treatment of patients pre-treated with platinum-containing chemotherapy	Disitamab vedotin 2.0 mg/kg IV once every 2 weeks (maximum dose 200 mg)
IMGN632	Breakthrough Therapy designation granted in October 2020	CD123/DNA mono-alkylating payload of the indolinobenzodiazepine pseudodimer (IGN) class.	NCT03386513	1/2	Relapsed or refractory or Untreated blastic plasmacytoid dendritic cell neoplasm (BPDCN)	IMGN632 IV
ARX788	Fast Track designation granted in January 2021	HER2/MMAF	NCT04829604 (ACE-Breast03)	2	HER2+ metastatic breast cancer, resistant or refractory to T-DM1, and/or T-DXd, and/or tucatinib-containing regimens	ARX788 IV Q4W

Abbreviations: MMAE: monomethyl auristatin E; Q3W: every 3 weeks (21 days); IV: intravenously; D: day(s); Q6W: every 6 weeks (42 days); HER2: human epidermal growth factor receptor 2; seco-DUBA: seco-duocarmycin-hydroxybenzamide-azaindole; RP2D: recommended phase 2 dose; QW: once per week; Q4W: every 4 weeks (28 days); NaPi2b: sodium-dependent phosphate transport protein 2B; MMAF: monomethyl auristatin F.

## Data Availability

Published data supporting this article are included within the reference list. Please contact corresponding author for any further requests or supplementary information.

## Abbreviations

ADCs	Antibody-drug conjugates
mAbs	Monoclonal antibodies
bsADCs	Bispecific ADCs
BSB	Binding-Site Barrier
PRLR	Prolactin receptor
GO	Gemtuzumab ozogamicin
BV	Brentuximab Vedotin
DAR	Drug-to-antibody ratio
T-DM1	Τrastuzumab emtasine
AML	Acute myeloid leukemia
Val-Cit	Valine-citrulline
PABC	Para-amino-benzyloxycarbonyl
PBDs	Pyrrolobenzodiazepines
TRX	1,2,4-trioxolane
uAA	Unnatural amino-acids
mTG	Microbial transglutaminase
SortA	Sortase A
FGE	Formylglycine-generating enzyme
V-ATPase	Vacuolar H^+^-ATPase
CPT	Camptothecin
AF-HPA	Auristatin F-hydroxypropylamide
MMAE	Monomethyl auristatin E
MMAF	Monomethyl auristatin F
TM-ADC	Trastuzumab–maytansinoid ADC
AAC	Antibody-antibiotic conjugate
MRSA	Methicillin-resistant *Staphylococcus aureus*
MSSA	Methicillin-susceptible *Staphylococcus aureus*
